# Effect of gabapentin on length of stay, opioid use, and pain scores in posterior spinal fusion for adolescent idiopathic scoliosis: a retrospective review across a multi-hospital system

**DOI:** 10.1186/s12871-022-01965-2

**Published:** 2023-01-07

**Authors:** De-An Zhang, Bruce Brenn, Robert Cho, Amer Samdani, Shriners Spine Study Group, Michelle Diu, Graham Fedorak, Purnendu Gupta, Matthew Kuestner, Cheryl Lawing, Scott Luhmann, Robert Moore, Sarah Oswald, Rolando Roberto, Casey Stondell, Vishwas Talwalkar, Pravin Taneja, Richard Vax, Polina Voronov, Michelle Welborn, Selina C. Poon

**Affiliations:** 1 Shriners Children’s Southern California, 909 S Fair Oaks Ave, Pasadena, CA 91105 USA; 2 Shriners Children’s Philadelphia, 3551 N Broad St, Philadelphia, PA 19140 USA

**Keywords:** Gabapentin, Gabapentinoid, Posterior spinal fusion, Adolescent idiopathic scoliosis, Pain, Opioid, Length of stay

## Abstract

**Background:**

Gabapentin has been adopted in Enhanced Recovery After Surgery protocols as a means to reduce opioid consumption while maintaining adequate post-operative analgesia. The purpose of our study was to review and compare changes in length of stay, opioid use, and patient reported pain scores after the addition of gabapentin into five, distinct pain protocols for posterior spinal fusion in adolescent idiopathic scoliosis.

**Methods:**

A retrospective review was completed using a database of electronic medical data from a single pediatric orthopedic healthcare system that was queried for patients with adolescent idiopathic scoliosis who underwent first-time posterior spinal fusion. Perioperative data including demographics, hospital length of stay, surgical details, opioid use, patient reported pain scores, and non-opioid analgesic use were collected.

**Results:**

From December 2012 to February 2019, 682 hospitalizations for posterior spinal fusion in adolescent idiopathic scoliosis were identified with complete inpatient data; 49% were administered gabapentin. For the gabapentin cohort, the system saw no statistically significant effect on length of stay or pain averaged over POD#0–3. Opioid use was statistically lower averaged over POD#0–3. Individual sites saw variation on length of stay and opioid use compared to the system.

**Conclusion:**

In conclusion, system-wide data showed gabapentin containing protocols reduced opioid use while maintaining clinically equivalent analgesia. However, variations of individual site results make it difficult to conclude the degree to which gabapentin were responsible for this effect.

**Supplementary Information:**

The online version contains supplementary material available at 10.1186/s12871-022-01965-2.

## Background

Enhanced recovery after surgery (ERAS) protocols were first published nearly 20 years ago. Since then, a multitude of guidelines have been published for a variety of surgical procedures. The underlying foundation of ERAS is to use a multidisciplinary and multimodal approach to minimize the stress response following surgery in order to optimize patient recovery [1]. One of the goals of ERAS protocols is to reduce the dependence on opioids for post-operative analgesia. Opioids carry several side effects including nausea, vomiting, sedation, pruritus, and urinary retention [2]. The American Society of Pain recommends gabapentinoids (gabapentin, pregabalin) be considered as part of a multi-modal approach to analgesia [3]. It is thought that gabapentinoids decrease acute post-operative pain by inhibiting the release of excitatory neurotransmitters from primary afferent nerve fibers thus reducing hyper-excitability of dorsal horn neurons following tissue injury during surgery [4].

Several studies have looked at the analgesic efficacy of gabapentin for posterior spinal fusion in adolescent idiopathic scoliosis. One study, which looked at the use of a single loading dose of gabapentin did not change post-operative pain or opioid use [5]. However, a series of four recent studies which used both a loading dose plus inpatient maintenance dosing showed deceases in opioid use and some improvement in post-operative pain [6–9].

In this study, we focus on the implementation of gabapentin as part of pain protocols in five geographically diverse, pediatric orthopedic institutions. Each autonomously managing their clinical protocols but operating under an umbrella healthcare system. We reviewed changes in length of stay, post-operative opioid use, and post-operative patient reported pain scores after the addition of gabapentin into pain protocols.

The purpose of our study is to compare the results at individual sites with the findings of the aforementioned studies. Our future goal is to use these findings to develop a standardized, system wide pain protocol.

## Methods

This study was reviewed by an Institutional Review Board and determined to be exempt as defined under federal regulation 45 CFR 46. A database containing de-identified electronic medical record data was queried from December 2012 to February 2019 for all patients with adolescent idiopathic scoliosis (AIS) who underwent first-time posterior spinal fusion (PSF). Data from nine, affiliated, pediatric orthopedic institutions across the United States, was returned. Pain protocols were designed and implemented independently at each site. This included the dosing protocol of gabapentin, use of other non-opioid analgesics, and opioid administration protocol. Sites using neuroaxial (intrathecal opioid or epidural infusions) anesthesia as part of their pain protocol were removed. Specific gabapentin dosing protocols during individual visits were not available. Table 1 provides the median loading and maintenance dose of gabapentin at each site to approximate the most common dosing protocol at each site.

**Table 1:** Median amount of gabapentin administered (mg/kg) per day at each site

Site	Load mg/kg/day	POD#1 mg/kg/day	POD#2 mg/kg/day	POD#3 mg/kg/day
Site 1	10.6	6.8	6.8	6.7
Site 2	4.7	10.1	13.4	12.4
Site 3	0	5.3	5.2	0
Site 4	9.3	10.3	13.3	3.7
Site 5	0	5.4	6.4	3.6

Perioperative data for this population; including demographics, hospital length of stay, surgical details, opioid use, patient reported pain scores, and non-opioid analgesic use; was collected. The primary outcome measures were length of stay, opioid use from post-operative day (POD) #0 to #3, and patient reported pain scores from POD#0 to POD#3.

Length of stay was calculated from the day of surgery to day of discharge. POD#0 was defined as arrival time in post-anesthesia care unit until midnight on the day of surgery. Subsequent post-operative days were defined as intervals from midnight to midnight. Opioid use (intravenous and oral) and pain scores (0–10 numeric rating system) were collected from POD#0 to POD#3. Individual patient opioid use was converted to oral morphine equivalents, summed, and normalized by weight for this time-period while pain scores were averaged. Statistical analysis we done using SPSS v27 (IBM Corporation, Armonk NY, USA). Fischer exact test and Mann–Whitney U test were used for non-normally distributed data. Statistical significance was defined as p < 0.05.

## Results

**Fig. 1:**
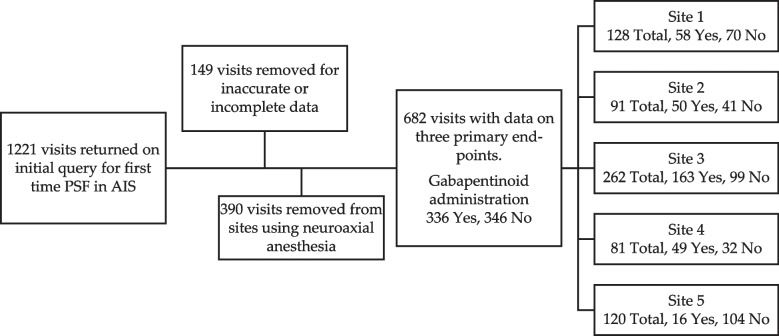
Initial query returned 1221 visits for first-time PSF in AIS. After review of data, 149 visits were removed due to inaccurate or incomplete data. Another 390 were removed from institutions using neuroaxial techniques as part of their pain protocol. Final study group consisted of 682 visits. Site-specific breakdown given with total number of visits followed by number of patients administered and not administered gabapentin during hospitalization

From December 2012 to February 2019, 682 hospitalizations for PSF in AIS were identified with complete inpatient data on length of stay, opioid use, and patient reported pain scores across the five sites (Fig. 1). For these 682 patients, 336 (49%) were administered gabapentin.

Table 2 provides demographic, surgical, and combined system data. The gabapentin administration group had statistically longer surgical times (Yes 409 min v No 394 min, *p* = 0.016) and more female patients (Yes 84% v No 77%, *p* = 0.033). The system combined gabapentin cohort saw no statistically significant effect on length of stay (Yes 5.2 v No 5.2, *p* = 1) or average pain (from POD#0–3 Yes 3.8 v No 3.9, *p* = 0.527). Opioid use was statistically lower averaged over POD#0–3 (Yes 4.2 v No 4.8, *p* < 0.001); with all days except POD#0 seeing a statistically significant decrease.

**Table 2:** Demographic, surgical, and results data for gabapentin and non-gabapentin groups across the system as a whole. For number of levels fused, unknown patients either lacked surgical billing codes or had non-specific codes. Pain score difference reports the average amount of decreased pain in the gabapentin group. Percent change in mean opioid use reports the percent decrease in opioid usage in the gabapentin group. Significance level of *p* < 0.05 via Fisher exact test and Mann–Whitney U test

	Gabapentin Administration			
	Yes	No	P	
Surgery Time (minutes)	409	394	0.016	
Gender (% female)	84%	77%	0.033	
Age (years)	14.7	14.9	0.222	
BMI	24.7	22.8	0.897	
Number of Levels Fused				
Unknown	3	5	0.386	
1–6	16	15		
7–12	202	227		
13 +	115	99		
Mean Length of Stay (days)	5.2	5.2	1	
Mean Average Pain from POD#0–3 (NIRS 0–10)	3.8	3.9	0.527	Difference 0.1
POD#0	3.6	4.1	0.067	0.5
POD#1	3.9	3.7	0.421	-0.2
POD#2	3.8	4.1	0.104	0.3
POD#3	3.8	3.9	0.405	0.1
Mean Opioid Use from POD#0–3 (Oral MME/kg)	4.2	4.8	< 0.001	Percent Change 14%
POD#0	0.8	0.8	0.582	0%
POD#1	1.5	1.7	0.004	13%
POD#2	1.1	1.3	< 0.001	18%
POD#3	0.9	1.0	0.021	11%

Demographic, surgical, and site-specific data for each of the three end-points is reported in Additional file 1. Site 4 saw a statistically different distribution in the number of levels fused with the gabapentin group having more surgeries of greater complexity and increased surgical time. The gabapentin group was younger in Site 3. Body mass index was greater for the gabapentin group in Site 2.

Three sites (Site 2, Site 3, Site 5) saw a statistically significant decrease in length of stay. Decrease in pain averaged over POD#0–3 was insignificant across all sites. Change in opioid usage was mixed. Three sites saw a statistically significant decrease (Site 1, Site 3, Site 5) in opioid use. Individual days across sites saw statistically significant fluctuations in both directions.

## Discussion

This was a single healthcare system, retrospective database review looking at the effect of gabapentin on hospital length of stay, total opioid use normalized to weight, and average patient reported pain across five different sites; each with a unique but similar pain protocol. Our results showed, when looking at combined system data, the addition of gabapentin to pain protocols decreased average opioid use from immediately after surgery until POD#3. No effect was seen on average pain or average length of stay. These findings on length of stay and opioid use were not reflected at each of the five sites.

When comparing our gabapentin findings with existing current literature, one must first examine the gabapentin dosing protocols in existing studies. Table 3 provides the gabapentin regimen for four existing studies.

**Table 3:** Gabapentin loading and maintenance dosing described in prior studies [6–9]. Calculated daily total dose (mg/kg) presented in *italics*

Study	Loading Dose	Maintenance Dose
Choudhry [6]	10 mg/kg up to 600 mg	Pt > 50 kg 200 mg TID Pt < 50 kg 100 mg TID ~ *6–12 mg/kg/day*
Rusy [7]	15 mg/kg	5 mg/kg TID *15 mg/kg/day*
Anderson [8]	15 mg/kg	10 mg/kg q8 hours *30 mg/kg/day*
Trzcinski [9]	10 mg/kg up to 600 mg	5 mg/kg TID, max 300 mg/dose *15 mg/kg/day, max 900 mg/day*

These studies had various protocols for loading dose between 10–15 mg/kg and maintenance dosing between 6 to 30 mg/kg/day [6–9]. Dosing protocols varied at our individual sites (Table 1), but median dosing trended lower than the described study protocols. These differences make comparing the efficacy of gabapentin difficult and points to the need for defining and standardizing an optimal gabapentin dosing protocol.

In looking at our three primary end-points, our system in total did not see a significant change in length of stay. This resembles Choudhry et al. finding no significant decrease in hours to discharge in the gabapentin group [6]. However, three sites (Site 2, Site 3, Site 5) saw gabapentin patients, on average, discharged sooner. In addition to pain control, several factors are responsible for hospital length of stay including ambulation, lack of surgical complications, and home readiness. We did not have information on discharge processes and criteria changes over time at our institutions. Without being to compare discharge criteria, it is difficult to conclude the effect of gabapentin on length of stay.

When considering effects on patient reported pain following surgery, it is important to first consider what a clinically significant change in pain score is. Research into pain scores has shown that in a 0–10 numeric rating scale (NRS) system, for patients experiencing moderate pain, a minimal change of 1.3 is needed by patients to report improvement [10]. Similarly in a 0–100 visual analogue scale (VAS) system, again for moderate pain (VAS of 31–70), a change of at least 10 points was deemed meaningful by patients [11]. In prior studies, only Rusy et al. reported a statistically and clinically significant decrease immediately following surgery (2.5 v 6) and first pain score the morning after surgery (3.2 v 5) [7]. In our data, the two groups had no clinically significant decrease averaged over POD#0–3 or on specific days. This lack of change in patient reported pain scores was not surprising. We had thought patients and nursing would maintain pain at the same tolerable levels before and after introduction of gabapentin. All previous studies showed gabapentin decreasing opioid usage approximately 30% with the effect greatest the first day or two after surgery [6–9]. In our system, the gabapentin group experienced a statistically significant decrease in opioid use averaged from POD#0–3 and each day except POD#1. However, only three sites saw a statistically significant decrease averaged over POD#0–3 (Site 1 14%, Site 2 25%, Site 5 20%). Daily statistically significant increases and decreases in opioid usage were seen scattered across the individual sites. When analyzing opioid use, it is important to consider if opioids were administered on a schedule, or if dosed as needed by patient request. Gabapentin groups may have required less opioids but were still administered opioids according to a scheduled protocol. Unfortunately, our database was unable to capture if opioids were given at a scheduled interval or as needed by patients. We hope to re-examine opioid administration protocols in the future as our database matures.

As a retrospective database review, there is an inherent limitation related to possible erroneously documented data in the electronic medical records. In an attempt to mitigate this effect, all data in this study was manually reviewed and outliers were discarded if unable to be corrected. Additionally, we lacked precise details on the degree of surgical correction, attempting to use duration of surgery and billing codes as proxy. Any changes in discharge processes over time at the individual institutions was unknown.

Further, variations in surgical technique approach and equipment likely exist in and between sites. These surgical variables should be considered in future multi-modal analgesic work.

Our five sites adopted different gabapentin dosing protocols to their underlying multi-modal pain protocols. This indicates, as prior studies have concluded, that more research needs to be done to determine the optimal gabapentin dosing protocol which balances analgesic potency with adverse side effects (sedation, dizziness, fall risk). Further, the efficacy of gabapentin needs to be studied in relation to other multi-modal analgesic techniques (scheduled/as need opioid dosing, methadone, non-opioid analgesics, neuroaxial anesthesia) in order to create an optimal multi-modal analgesic protocol.

## Conclusion

Our system-wide data suggests the addition of gabapentin to multi-modal pain protocols can decrease the use of opioids while maintaining clinically equivalent analgesia. The heterogeneity in gabapentin dosing and underlying pain protocols, along with the varying individual site results, makes it difficult to conclude the degree to which gabapentin were responsible for this effect. Our more pertinent finding is the need for institutions to verify the desired clinical effect of new or modified components of multi-modal analgesic protocols by defining, collecting, and analyzing objective metrics of quality improvement.

## Supplementary Information

**Additional file 1:** Demographic, surgical, and outcome data for each individual site is presented.

## Data Availability

Restrictions apply to the availability of these data. Data are available upon request from the corresponding author for researchers who meet the criteria for access to confidential data.

## Abbreviations

ERAS: Enhanced recovery after surgery
AIS: Adolescent idiopathic scoliosis
PSF: Posterior spinal fusion
POD: Post-operative day
NRS: Numeric rating scale
VAS: Visual analogue scale
