# Supplementary material for: Effect of gabapentin on length of stay, opioid use, and pain scores in posterior spinal fusion for adolescent idiopathic scoliosis: a retrospective review across a multi-hospital system
Source: BMC Anesthesiol. 2023 Jan 7;23:10. doi: 10.1186/s12871-022-01965-2 (PMC9824993; doi:10.1186/s12871-022-01965-2)
Supplement: Supplementary file 1 — Additional file 1: Demographic, surgical, and outcome data for each individual site is presented. [file 12871_2022_1965_MOESM1_ESM.docx]

**Appendix A**

|  | Surgery Time (minutes) | | | Gender (% female) | | | Age (years) | | | BMI | | |
| --- | --- | --- | --- | --- | --- | --- | --- | --- | --- | --- | --- | --- |
| Site | Yes | No | p | Yes | No | p | Yes | No | p | Yes | No | p |
| Site 1 | 416 | 410 | 0.730 | 86 | 71 | 0.054 | 14.7 | 14.9 | 0.518 | 25.4 | 21.5 | 0.087 |
| Site 2 | 425 | 440 | 0.249 | 84 | 83 | 0.864 | 14.6 | 14.7 | 0.979 | 25.5 | 23.2 | **0.045** |
| Site 3 | 408 | 392 | 0.593 | 80 | 83 | 0.792 | 14.7 | 16 | **0.023** | 23.1 | 24.9 | 0.569 |
| Site 4 | 371 | 335 | **0.009** | 80 | 75 | 0.785 | 14.7 | 14 | 0.216 | 23.4 | 21.8 | 0.273 |
| Site 5 | 340 | 359 | 0.497 | 94 | 74 | 0.114 | 14.7 | 14.9 | 0.838 | 22.3 | 22.8 | 0.629 |

**Table A1.** Individual site specific demographic differences between gabapentinoid and non-gabapentinoid groups at each site and overall. Significance level of p < 0.05 via Fisher exact test and Mann-Whitney U test.

|  | Number of Levels Fused | | | | | | | | |
| --- | --- | --- | --- | --- | --- | --- | --- | --- | --- |
|  | Unknown | | 1-6 | | 7-12 | | 13+ | |  |
|  | Gabapentinoid Use | | | | | | | |  |
| Site | Yes | No | Yes | No | Yes | No | Yes | No | p |
| Site 1 | 0 | 3 | 5 | 3 | 28 | 30 | 25 | 34 | 0.278 |
| Site 2 | 3 | 1 | 5 | 3 | 102 | 65 | 53 | 30 | 0.926 |
| Site 3 | 0 | 0 | 2 | 3 | 40 | 34 | 8 | 4 | 0.565 |
| Site 4 | 0 | 0 | 4 | 5 | 23 | 21 | 22 | 6 | **0.049** |
| Site 5 | 0 | 1 | 0 | 1 | 9 | 77 | 7 | 25 | 0.401 |

**Table A2.** Individual site specific differences in number of levels fused in gabapentinoid and non-gabapentinoid cohorts. Unknown patients either lacked surgical billing codes or had non-specific codes. Significance level of p < 0.05 via Fisher exact test.

|  | Mean Length of Stay (days) | | |  |
| --- | --- | --- | --- | --- |
| Site | Yes | No | p | Difference |
| Site 1 | 5.2 | 5.3 | 0.420 | 0.1 |
| Site 2 | 5.8 | 6.2 | **0.021** | 0.4 |
| Site 3 | 4.5 | 5.5 | **< 0.001** | 1 |
| Site 4 | 4.2 | 4.0 | 0.434 | -0.2 |
| Site 5 | 4.2 | 4.4 | **0.020** | 0.2 |

**Table A3.** Individual site specific differences in effect of gabapentinoids on mean length of stay. Positive difference signifies reduction in length of stay in the gabapentinoid group while negative difference signifies a longer length of stay. Significance level of p < 0.05 via Mann-Whitney U test.

|  | Mean Average Pain  from POD#0-3 (NIRS 0-10) | | | |  |  |  |  |
| --- | --- | --- | --- | --- | --- | --- | --- | --- |
| Site | Yes | No | p | Difference |  |  |  |  |
| Site 1 | 4.5 | 4.9 | 0.050 | 0.4 |  |  |  |  |
| Site 2 | 3.5 | 3.4 | 0.720 | -0.1 |  |  |  |  |
| Site 3 | 3.8 | 4.1 | 0.629 | 0.3 |  |  |  |  |
| Site 4 | 3.9 | 3.4 | 0.189 | -0.5 |  |  |  |  |
| Site 5 | 3.3 | 38 | 0.288 | 0.5 |  |  |  |  |
|  | Mean Average Pain POD#0 | | | | Mean Average Pain POD#1 | | | |
| Site | Yes | No | p | Difference | Yes | No | p | Difference |
| Site 1 | 4.8 | 5.4 | **0.038** | 0.6 | 4.5 | 4.8 | 0.200 | 0.3 |
| Site 2 | 2.5 | 3.4 | 0.058 | 0.9 | 3.8 | 3.3 | **0.023** | -0.5 |
| Site 3 | 4.3 | 4.3 | 0.896 | 0.0 | 3.4 | 3.4 | 0.520 | 0.0 |
| Site 4 | 3.6 | 3.6 | 0.963 | 0.0 | 3.7 | 3.3 | 0.215 | -0.4 |
| Site 5 | 3.6 | 3.8 | 0.660 | 0.2 | 3.4 | 3.7 | 0.557 | 0.3 |
|  | Mean Average Pain POD#2 | | | | Mean Average Pain POD#3 | | | |
| Site | Yes | No | p | Difference | Yes | No | p | Difference |
| Site 1 | 4.5 | 4.9 | 0.057 | 0.4 | 4.2 | 4.6 | 0.072 | 0.4 |
| Site 2 | 3.5 | 3.9 | 0.132 | 0.4 | 3.5 | 3.4 | 0.785 | -0.1 |
| Site 3 | 3.8 | 4.1 | 0.317 | 0.3 | 3.8 | 4.1 | 0.677 | 0.3 |
| Site 4 | 4.1 | 3.7 | 0.464 | -0.4 | 4.1 | 3.7 | 0.319 | -0.4 |
| Site 5 | 3.6 | 3.8 | 0.517 | 0.2 | 3.5 | 3.8 | 0.330 | 0.3 |

**Table A4.** Individual site specific differences in effect of gabapentinoids on average pain (NRS 0-10). Positive difference signifies reduction in gabapentinoid group pain whereas negative difference signifies increase in gabapentinoid group pain. Significance level of p < 0.05 via Mann-Whitney U test.

|  | Mean Opioid Use from POD#0-3 (Oral MME/kg) | | | |  |  |  |  |
| --- | --- | --- | --- | --- | --- | --- | --- | --- |
| Site | Yes | No | p | % Change |  |  |  |  |
| Site 1 | 5.1 | 5.9 | **0.024** | 14% |  |  |  |  |
| Site 2 | 3.6 | 3.6 | 0.799 | -1% |  |  |  |  |
| Site 3 | 5.6 | 7.5 | **< 0.001** | 25% |  |  |  |  |
| Site 4 | 3.6 | 3.7 | 0.985 | 2% |  |  |  |  |
| Site 5 | 3.5 | 4.4 | **0.015** | 20% |  |  |  |  |
|  | Mean Opioid Use POD#0 | | | | Mean Opioid Use POD#1 | | | |
| Site | Yes | No | p | % Change | Yes | No | p | % Change |
| Site 1 | 0.9 | 1.0 | 0.300 | 10% | 1.7 | 2.2 | **0.004** | 21% |
| Site 2 | 0.5 | 0.3 | **< 0.001** | -63% | 1.2 | 1.0 | **< 0.001** | -28% |
| Site 3 | 1.5 | 1.6 | 0.503 | 6% | 2.0 | 2.8 | **0.002** | 27% |
| Site 4 | 0.5 | 0.5 | 0.721 | -4% | 1.3 | 1.2 | 0.743 | -5% |
| Site 5 | 0.9 | 0.9 | 0.829 | 3% | 1.3 | 1.8 | **0.050** | 24% |
|  | Mean Opioid Use POD#2 | | | | Mean Opioid Use POD#3 | | | |
| Site | Yes | No | p | % Change | Yes | No | p | % Change |
| Site 1 | 1.3 | 1.6 | **0.004** | 18% | 1.2 | 1.1 | 0.918 | -5% |
| Site 2 | 1.0 | 1.2 | **0.002** | 15% | 0.9 | 1.2 | **< 0.001** | 24% |
| Site 3 | 1.3 | 2.1 | **< 0.001** | 36% | 0.8 | 1.1 | **0.001** | 31% |
| Site 4 | 1.1 | 1.3 | 0.265 | 11% | 0.7 | 0.7 | 0.820 | 3% |
| Site 5 | 0.8 | 1.0 | 0.056 | 17% | 0.4 | 0.7 | **0.001** | 38% |

**Table A5.** Individual site specific differences in effect of gabapentinoids on opioid use (Oral MME/kg). Positive percent change signifies reduction in gabapentinoid group opioid use whereas negative percent change signifies increase in gabapentinoid group opioid use. Significance level of p < 0.05 via Mann-Whitney U test.
